# How to identify peer-reviewed publications: Open-identity labels in scholarly book publishing

**DOI:** 10.1371/journal.pone.0214423

**Published:** 2019-03-25

**Authors:** Emanuel Kulczycki, Ewa A. Rozkosz, Tim C. E. Engels, Raf Guns, Marek Hołowiecki, Janne Pölönen

**Affiliations:** 1 Scholarly Communication Research Group, Adam Mickiewicz University in Poznań, Poznań, Poland; 2 Centre for R&D Monitoring (ECOOM), Faculty of Social Sciences, University of Antwerp, Antwerp, Belgium; 3 Federation of Finnish Learned Societies, Helsinki, Finland; Universitat de Valencia, SPAIN

## Abstract

This article discusses the open-identity label, i.e., the practice of disclosing reviewers’ names in published scholarly books, a common practice in Central and Eastern European countries. This study’s objective is to verify whether the open-identity label is a type of peer-review label (like those used in Finland and Flanders, i.e., the Flemish part of Belgium), and as such, whether it can be used as a delineation criterion in various systems used to evaluate scholarly publications. We have conducted a two-phase sequential explanatory study. In the first phase, interviews with 20 of the 40 largest Polish publishers of scholarly books were conducted to investigate how Polish publishers control peer reviews and whether the open-identity label can be used to identify peer-reviewed books. In the other phase, two questionnaires were used to analyze perceptions of peer-review and open-identity labelling among authors (*n* = 600) and reviewers (*n* = 875) of books published by these 20 publishers. Integrated results allowed us to verify publishers’ claims concerning their peer-review practices. Our findings reveal that publishers actually control peer reviews by providing assessment criteria to reviewers and sending reviews to authors. Publishers rarely ask for permission to disclose reviewers’ names, but it is obvious to reviewers that this practice of disclosing names is part of peer reviewing. This study also shows that only the names of reviewers who accepted manuscripts for publication are disclosed. Thus, most importantly, our analysis shows that the open-identity label that Polish publishers use is a type of peer-review label like those used in Flanders and Finland, and as such, it can be used to identify peer-reviewed scholarly books.

## Introduction

Extant studies on peer-review practices have focused mostly on journal articles or grant proposals [1, 2]. Giménez-Toledo et al. [3] argue that few studies exist that examine peer reviews of scholarly publications, i.e., monographs, edited volumes, and chapters. Many practices–e.g., avoiding conflicts of interest–are common, in peer reviews of both journal articles and published scholarly books. However, the latter may be conducted in a different way than the former and may be driven by different values. Publishers of scholarly books that publish both original research and books for wider audiences (e.g., student textbooks), in peer reviews, not only use scholarly criteria, like originality of results, but also consider some commercial criteria, including target audience or unique selling points [4].

The peer-review process is an important control mechanism in the academic community. The control is realized through academic peers who judge the research quality of manuscripts, grant proposals, and other such texts [5, 6, 7]. However, publishers and editors ultimately control peer review process [8]. They organize the entire process, choose peers, manage bias, and guard against conflicts of interest. Thus, publishers’ decisions and how they control and organize this process shape, to some extent, whether a particular peer-review process accomplishes the task entrusted to it, i.e., accepting and improving good manuscripts, and rejecting poor ones.

Peer review of scholarly books before publication has become a delineation criterion in various systems used to evaluate scholarly publications. For instance, in Denmark, Flanders (Belgium), Norway, Spain, and Poland, academic books must be peer-reviewed to secure inclusion in performance-based research-funding systems. In Finland, scholarly publications that have been peer-reviewed carry more weight than non-peer-reviewed monographs, chapters, and edited works [3, 9].

Evaluation practices for scholarly books differ across countries and take various forms [10, 11, 12]. In Denmark, Finland, Flanders, Norway, and Spain, authoritative lists of book publishers and/or book series are used. The value of scholarly monographs, chapters, and edited volumes in universities’ funding schemes depend on the outlets indicated on those lists. Until 2017, in Poland, scholarly books were valued only when they met various technical criteria, including a minimum book length of 240,000 characters. In Finland and Flanders, in addition to lists of publishers, specific peer-review labels are used to identify peer-reviewed scholarly publications.

The Federation of Finnish Learned Societies (TSV) developed the peer-review label in Finland, i.e., the label for peer-reviewed scholarly publications, to help scholarly publishers indicate which of their published books and articles have gone through peer review (https://tsv.fi/en/services/label-for-peer-reviewed-scholarly-publications). Publishers can apply for the right to use the peer-review label, which TSV administrators grant. The publishers agree to a series of requirements concerning the peer-review process, its documentation, and printing of the label in publications. Publishers also must provide detailed information on their websites concerning the peer-review process and state their commitment to TSV label standards. Scholarly publications must be peer-reviewed prior to publication by at least two independent experts, and the process must focus on the manuscript’s scientific quality according to the pertinent field’s standards. The publisher also must send referees’ reports to the authors of submissions. The label can be affixed to monographs, as well as individual chapters and articles in books or journals. The label also is independent of the authority list of publication channels used in the performance-based research funding system, and output from scholarly publishers who do not use the label is considered in the funding scheme. However, the label supports the overall data-collection process by helping researchers and other involved personnel identify peer-reviewed monographs and articles registered with local current research information systems. Indeed, if a publisher uses the label properly, only monographs, chapters, and edited works containing the identifier are reported as peer-reviewed publications. The publishers–mostly learned societies–are entrusted to use the label according to the requirements, with no regular reporting of required documentation to TSV necessary. Compliance with the requirements is investigated only if a complaint is lodged, which may result in a publisher losing the right to use the label. Also, the National Board of Research Integrity may request documentation from publishers in the event of misconduct investigations. Currently, 10 book publishers and 173 journals and book series use the label.

The peer-review label in Flanders, i.e., the Guaranteed Peer Reviewed Content (GPRC) label, is a quality label that the Flemish Publisher Association initiated, and the Group of Educational and Scientific Publishers currently maintains it. The label indicates that a publication has been subjected to peer review by two reviewers in a peer-review process corresponding with international academic standards [13, 14]. While the label was conceived as a reaction to changes in the Flemish funding system, specifically the introduction of the VABB-SHW database for social sciences and humanities publications [15], it is not directly tied to it. The panel that oversees the inclusion of publications in the VABB-SHW may still check some GPRC-labeled books and–in rare cases–decide not to include them. Finland and Flanders’ peer-review labels are represented by graphic marks that indicate a given text was peer-reviewed. The labels can be placed on editorial pages, book covers, or, in the case of Finland, on table-of-contents pages and on individual papers.

Three principal attributes characterize Finland and Flanders’ peer-review labels: (1) a label serves as evidence that a peer review was conducted, (2) the publisher controls the peer-review process, and (3) the publisher archives all documents and reviews.

In this study, we investigate a common practice that has been widespread for several dozen years in Central and Eastern European countries: open identities in scholarly book publishing. *Open identity* is a term related to *open review* as a form of peer review and means that reviewers’ names are published in the books that they review [16, 17]. In Central and Eastern European nations, scholarly publishers commonly disclose the names of those who review published scholarly books. Generally, this information is presented on the editorial page and sometimes on the back cover of the book, where quotes from reviews often are presented. Moreover, reviewers’ scientific degrees, titles, and affiliations might be provided, along with reviewers’ names. In this study, we focus on *open identity* as practiced in scholarly books published in Poland. It is noteworthy that this is a social practice, not a formally operated standard, like the peer-review labels in Finland and Flanders. Nonetheless, in Latvia and Ukraine, for instance, some regulations require that monographs undergo reviews, with reviewers’ names disclosed. In Latvia, the Latvian Council of Science has a procedure in place to grant expert rights to scientists. One of the quality criteria in the assessment process in social sciences and humanities is a scholarly monograph that a candidate publishes to demonstrate expert abilities. In a monograph, reviewers’ names must be disclosed, and reviewers cannot represent the same institution as the monograph’s author (Procedure for granting Latvian Council of Science expert rights. Written Procedure Decision Nr. 19-1-1 January 15, 2018). Ukraine has a requirement regarding monographs submitted as dissertations for degrees of doctor and candidates of sciences. Such a monograph must contain the following information about reviewers: at least two doctors of sciences who are experts in the dissertation’s specialty (requirements for the published monograph, submitted for the degree of doctor and candidate of sciences, November 2, 2012, 1852/22164).

For purposes of this study, we coined the term *open-identity label*, in which reviewers’ names are disclosed, for instance, on the editorial page or on a book cover.

This study’s principal objective is to address the following research question: Does the *open identity label* used for scholarly publications operate as a type of peer-review label? On the basis of our research question and the aforementioned three attributes characterizing peer-review labelling, we have formulated the following statements:

S1: Open-identity labels confirm that monographs have been peer-reviewed.
S2: Ph.D.-level researchers or other experts from the pertinent field peer-review manuscripts.
S3: Publishers give reviewers explicit evaluation criteria.
S4: Publishers send reviews to authors.
S5: Publishers ask the reviewers for permission to publish their names.
S6: Publishers archive the documents produced during the peer-review process.

To verify these statements, then address the principal research question, we investigated how Polish publishers of scholarly books accept manuscripts for publication and conduct peer reviews. Moreover, we focused on the practice of disclosing reviewers’ names on monographs’ editorial pages; thus, our approach highlights current editorial practices.

## Materials and methods

We have conducted a mixed-method sequential explanatory study [18] comprising semi-structured interviews and statistical analyses to investigate publishers’ perspectives on their own practices and to verify publishers’ claims concerning their peer-review practices. This method allows us to investigate whether Polish publishers control the peer-review process, i.e., whether they provide reviewers with evaluation criteria, send reviews to authors, and archive the documents created in the process. Thus, in this study, examining whether publishers control the peer-review process is achieved via triangulation of publishers’ claims and authors and reviewers’ perceptions.

This study comprises two phases. In the first phase, we interviewed 20 of the largest Polish scholarly book publishers, which were selected according to the number of scholarly books published during the 2013–2016 period. In the second phase, we conducted two surveys. Questionnaire 1 was used to analyze the perceptions of monograph authors whose monographs were published by these 20 publishers, and Questionnaire 2 was used to analyze the perceptions of reviewers who evaluated the monographs that these 20 publishers published, specifically how they viewed the peer-review process. The second phase allowed us to triangulate the results and verify how publishers’ claims are related to authors and reviewers’ perceptions.

At the beginning of interviews, we informed interviewees about the study objectives, study authors, and we confirmed that all retrieved information is confidential. We asked for permissions to record interviews, transcribe them, and use anonymized excerpts to illustrate editorial practices. We asked for oral approvals and recorded them. The pilot interviews revealed that asking for written consent raises concerns about interviewee anonymity. Additionally, in the welcome messages of questionnaires, we have communicated that the survey is fully anonymous thanks to using tokens generated by LimeSurvey (https://www.limesurvey.org). Such a procedure has allowed us to not seeking approval of the ethics committee because we have not obtained any sensitive personal data and the data were analyzed anonymously.

We used three datasets: (A) to present publishers’ peer-review claims and analyze their practices; (B1) to determine whether the monographs’ authors confirm that these peer-review practices that publishers presented are used (S1 File); and (B2) to determine whether reviewers confirm that publishers’ peer-review practices are used. The three datasets that we used are described below (S2 File).

### Dataset A

We conducted 20 semi-structured interviews with the directors of publishing houses or other who are responsible for the book-evaluation process in a given publishing house. We chose publishers according to a ranking of the Polish publishers based on the number of scholarly books published during the 2013–2016 period. The National Library in Poland provided the data used in the ranking process. To secure publishers’ anonymity, we randomly selected 20 of the 40 largest publishers in terms of number of scholarly books published between 2013 and 2016. We used proportionate stratified sampling, in which publisher profiles (university publisher/commercial publisher) are the stratum. In the final dataset, we analyzed 15 university publishers and five commercial publishers. The mean number of scholarly books published during the 2013–2016 period is 320 among the university publishers and 498 among the commercial ones. These 20 publishers published 18.5% of the total number of monographs accepted in the national research exercise in 2017 for the 2013–2016 period.

The objective in conducting the interviews was to test generated statements (S1–S6) related to controlling the peer-review process. We also conducted a pilot interview with a publisher (not included in the interviews with these 20 publishers). On the basis of the pilot results, we improved the sample questions. The final interviews were conducted in Polish by one of the present study’s authors. The average interview length was approximately 54 minutes. We asked the interviewees about their practices, relations with authors and reviewers, and formalized regulations for book evaluations (topics and sample questions were translated into English and provided in Appendix 1).

### Dataset B1

We sent an online survey to the authors of monographs published by 20 publishers from Dataset A. The survey’s objective was to determine whether authors whose monographs were published by these publishers would confirm that the publishers used the peer-review practices that they espoused. We used bibliographical information on the peer-reviewed monographs collected within the Polish performance-based research funding system in Poland [11]. On the basis of organizational classification used in this system, we assigned one of six fields of science and technology, designed by the Organization for Economic Cooperation and Development (OECD), to each monograph. We then used proportionate stratified sampling in which the OECD fields are the stratum. We assumed that peer reviews of monographs assigned to the different OECD fields do not vary generally, but that differences in details may exist (e.g., evaluation criteria). We randomly selected 600 single-authored monographs (30 monographs per publisher) published during the 2013–2016 period. Using authors’ names, we searched for their email addresses (institutional addresses were preferred) on university websites, the Polish database on researchers (“Ludzie Nauki” [http://nauka-polska.pl]), and other resources, e.g., academic blogs, emails provided in the journal articles, etc. In the event that we were unable to connect an email address to an author (*n =* 232), we randomly selected other monographs for the dataset, while maintaining the same sampling method. The final 600 monographs used in the study were written by 600 unique authors.

### Dataset B2

We sent an online survey to reviewers of these monographs, which were used to select authors for Dataset B1. The survey’s objective was to determine whether reviewers who reviewed monographs chosen in Dataset B1 would confirm that the publishers actually used the peer-review practices that they espoused.

Having bibliographic information on monographs and authors’ names, we consulted the monograph copies (with the overwhelming majority from university libraries) and noted reviewers’ names from the editorial pages of these 600 monographs. Moreover, when the data on reviewers’ affiliations and degrees were available, these data also were written down. On the basis of this information, we searched for reviewers’ email addresses in the same way we did for authors.

Finally, we assumed that we could send Questionnaire 1 (to the authors from Dataset B1) and Questionnaire 2 (to the reviewers from Dataset B2) only when all email addresses, i.e., those of the authors and reviewers of a given monograph, are available. Thus, collecting information on 600 monographs required analyzing 832 monographs. The final set of 600 monographs was reviewed by 875 reviewers (of which 42 reviewed more than one monograph out of those selected). Monographs were reviewed either by a single reviewer (*n* = 325), two reviewers (*n* = 268), or three or more reviewers (*n* = 7). The mean number of reviewers per monograph was 1.47.

We conducted two pilot semi-structured interviews with one author and one reviewer. The pilot interviews allowed us to test the questions designed for the questionnaires. Moreover, we sent Questionnaire 1 to one author and Questionnaire 2 to one reviewer to test the questionnaires.

To send out the anonymous surveys, we used the online tool LimeSurvey to send out 40 questionnaires: one survey for authors per publisher and one survey for reviewers per publisher. We asked the authors about the peer-review process for their books and reviewers’ comments (see the questionnaire translated into English in Appendix 2). We asked the reviewers about the peer-review process, relations with publishers, and whether publishers asked for permission to disclose their names (see the questionnaire translated into English in Appendix 3). Finally, we received 177 fully completed questionnaires from the authors and 212 from the reviewers. The overall response rate to the surveys was 33% (Questionnaire 1) and 28% (Questionnaire 2) after excluding bounced emails (72 and 116, respectively).

### Data analysis

We analyzed and integrated the quantitative and qualitative data using the *statement (hypothesis) coding approach* [19, 20] in five steps described in detail below. Results are presented according to the *weaving approach*, which enables us to present both qualitative and quantitative results together on a theme-by-theme basis [21, 22].

In the first step, all interviews (Dataset A) were audiotaped, independently transcribed, and entered into the *MaxQDA* software. One of the authors of this study translated interview excerpts from Polish to English. The initial coding scheme was determined by statements (S1–S6). We coded the data with an emphasis on publishers’ attitudes in controlling the peer-review process. According to the *statement coding approach*, we aim to find such statements in transcribed interviews that confirm or deny the statements in the hypotheses. Coded statements have allowed us to verify whether particular statements (S1–S6) are confirmed for a given publisher. Dataset A served to provide both an in-depth understanding of how the peer-review process is controlled and to expand Datasets B1 and B2 by adding new variables.

In the second step, for each publisher from Dataset A, we constructed six new variables related to tested statements (S1–S6). Each of these variables has one of two values regarding whether a particular statement is confirmed (value 1) or not (value 2) on the basis of verification conducted in the first step of this analysis.

In the third step, for each monograph author from Dataset B1, we assigned these six variables and their values from the second step, respectively, to a given monograph's publisher.

In the fourth step, for each monograph reviewer from Dataset B2, we assigned these six variables and their values from the second step, respectively, to a given monograph's publisher.

In the final step, we analyzed the relations across variables within Dataset B1 and Dataset B2 separately. We also compared authors’ responses with publishers’ claims, and reviewers’ responses with publishers’ claims on controlling the peer-review process by testing relations among variables related to these responses and claims. The final analysis was conducted with IBM SPSS Statistics software (ver. 24).

We combined the results of the qualitative phase (step 1) and quantitative phase (steps 2–5) of this study and presented them together using the *weaving approach*. Presenting the results from testing each statement, we started to describe the results based on Dataset A. We illustrated them through excerpts from interviews (each excerpt is identified by a separate code to keep the publisher anonymous, e.g., “E11”). Then we presented the results from testing relations among variables related to these authors and reviewers’ responses and publishers’ claims, allowing us to verify whether publishers’ claims corresponded with authors and reviewers’ statements.

## Results

### S1: Open-identity labels confirm that monographs were peer-reviewed

In this analysis, we assumed that disclosing reviewers’ names on monographs’ editorial pages confirms that given monographs were peer-reviewed. According to all analyzed publishers, this assumption is fully justified. One publisher noted: “In this way, we confirm that a book was peer-reviewed, and it is a scholarly book” (E11). Another stated that disclosing reviewers’ names “is a matter of co-responsibility for the scientific character [of a given monograph]; the name and the reviewer’s scientific degree are some kind of confirmation of the publication quality” (E3).

The open-identity label is understood to be a type of certificate that Polish publishers of scholarly books commonly use, even though no legal obligation exists requiring disclosure of reviewers’ names. Some publishers believe that the open-identity label is a prerequisite to acknowledge about a given book as a scholarly publication that could be counted in research-evaluation systems: “Currently, this [disclosing reviewers’ names] results from some formal criteria. A book must be peer-reviewed to be acknowledged as a monograph and be counted in the Polish research-evaluation system. So, we mark who reviewed a book on the editorial page” (E1); “it is an obligation (…) even a good monograph cannot be used for academic promotion when there is no information about reviewers. It is a fundamental condition” (E19).

Authors’ responses confirm publishers’ claims, with 170 of 177 authors (96%) confirming that their monographs were peer-reviewed. Seven authors (4%) denied or were unsure whether their manuscripts were peer-reviewed. Six publishers published these seven authors’ monographs; thus, these were outliers and not a general trend. Therefore, it is justified to say that the authors confirmed that open-identity labels are products of the peer-review process.

We received emails from nine reviewers claiming that they could not complete our survey because they did not remember whether they reviewed any books for a particular publisher. We informed each reviewer about the monograph in which his or her name is disclosed as a monograph reviewer. Moreover, we attached a photo of the editorial page as evidence. Finally, four of nine reviewers confirmed reviewing given monographs, with two claiming to have reviewed only a Ph.D. thesis manuscript, as they did not know that the thesis was published as a monograph. Three reviewers did not reply to our emails. None of the 875 reviewers to whom we sent emails denied that they participated in the reviewing process.

### S2: Ph.D.-level researchers or other experts from pertinent fields peer-reviewed manuscripts

We assumed that Ph.D.-level researchers or experts from the field in which a given monograph was published reviewed the manuscripts. All publishers claimed that they always ask for reviewers who are at least Ph.D.-level researchers. In Poland, two scientific degrees are bestowed: Ph.D.s and the highest degree: habilitation. Therefore, from publishers’ perspective, the most welcome reviewers are researchers who hold a habilitation degree or are full professors. The following statements illustrate this view: “In general, monographs are reviewed by older peers who hold a habilitation, possibly outstanding experts who are Ph.D.-level researchers” (E171); “only researchers with habilitation have the right to review monographs for us" (E194). Most publishers (*n* = 16) stressed that reviewers should be experts in a given field: “These must be researchers who are experts in the field in question” (E190); “a reviewer needs to do research in a given field” (E165).

In choosing reviewers, publishers also vigilantly watch out for potential conflicts of interest. Most publishers (*n* = 17) stressed that they seek reviews only from so-called “external reviewers.” This term very rarely was defined, but many of the university publishers understood *external reviewers* to be reviewers that a given university does not employ: “Reviewers cannot be staff or retired staff members of our university” (E167).

All reviewers who responded to our survey held habilitation degrees when they reviewed the monographs we asked about. This confirms publishers’ claims that they ask for reviews only from researchers who hold habilitation degrees. The overwhelming majority of reviewers assessed their own qualifications for reviewing given manuscripts as very high or high (*n* = 200; 94%), nine reviewers (4%) self-assessed their qualifications as medium, and only three reviewers (1%) assessed their credentials as low or very low. Reviewers’ responses confirm that publishers pay attention to the fields that reviewers represent and to their expertise levels.

### S3: Publishers give reviewers explicit evaluation criteria

We assumed that publishers using open-identity labels control the peer-review process by giving reviewers evaluation criteria. Publishers’ claims indicated that this practice exists among 13 of 20 publishers. These publishers provide criteria as part of the agreement or as a separate review form: “Manuscripts are reviewed according to a form provided to reviewers” (E39); “in the agreement, we state what are our expectations from reviews (…) reviews need to be comparable, so that it is not the case that someone writes only ‘I like the book; you should publish it’” (E6).

The seven publishers that do not give reviewers evaluation criteria believe that they should not influence the peer-review process through evaluation criteria. One of these publishers told us: “We don't have special expectations. I think that well-educated people who hold scientific degrees know what a review should look like” (E36). Some publishers indicate that the reviewers take full responsibility for the reviews: “We leave criteria to the experts, who should be responsible for the scope of a given book” (E56); “in general, from the publisher’s perspective, the author and the reviewers who signed the reviews are responsible for the content of monographs. Therefore, we do not set any restrictions” (E58). Publishers that do not provide evaluation criteria include both the majority of commercial publishers (i.e., three out of five) and a minority of university publishers (i.e., four of 15). Therefore, this is not a feature that could help differentiate between both types of publishers.

We compared two perspectives, i.e., reviewers’ responses and publishers’ claims on providing evaluation criteria. Table 1 presents reviewers’ responses to the question of whether the publisher provided evaluation criteria. These responses are correlated with corresponding publishers’ claims.

**Table 1:** A comparison of reviewers’ responses (*n* = 212) on whether publishers provide evaluation criteria, along with corresponding publishers’ claims.

	Number of publishers	Publishers’ perspective	Reviewers’ perspective				Total number of responses	
			Provided		Not provided			
			n	%	n	%	n	%
	13	Provided	70	49	73	51	143	100
	7	Not provided	31	45	38	55	69	100
Total	20	–	101	48	111	52	212	100

As Table 1 shows, 101 (48%) of reviewers received criteria from publishers, whereas 111 (52%) of reviewers did not receive any criteria. Thus, publishers’ claims do not correspond with reviewers’ responses. Receiving evaluation criteria from publishers is not related significantly to publishers’ claims on providing such criteria (χ^2^ = .58, *p* > .05). Only 49% of reviewers who reviewed monographs for publishers who claimed that they provided criteria responded that they received evaluation criteria. At the same time, a large percentage of reviewers (45%) who reviewed monographs for publishers who claimed that they did not provide criteria responded that they actually received such criteria. This may indicate that within both groups of publishers (claimed providing or not providing criteria) a substantial arbitrariness exists in how they cooperate with reviewers. However, one can also surmise that much potential variation exists in providing criteria, ranging from a general description (e.g., “please address the quality of writing and scientific rigor in your review to a fill-out form”). Thus, researchers and publishers may interpret this issue differently.

### S4: Publishers send reviews to authors

We assumed that publishers using open-identity labels control the peer-review process by sending reviews to authors, making this process transparent for authors. Moreover, reviews allow publishers to reject poor manuscripts, as well as approve and improve good ones.

All interviewed publishers claimed that they send reviews to authors. Describing this process, many publishers focused on what is actually sent: “When we get a review, of course, we read it, we analyze it, and inform the author that a review is ready, and we just send it to the author” (E92); “we send to the authors the reviews, conclusions, (and) sometimes there are also comments on the printout of (the) manuscript, which we (send) to the reviewer. So, this whole package is sent to the author” (E87). Other publishers emphasized that reviews serve as a way to help authors refine and improve their manuscripts: “Reviews are sent to the authors because they work with them” (E74). Publishers also stressed that authors should revise manuscripts and provide responses for reviewers: “The author gets the review, naturally without the reviewer’s name (…) and the author must address the reviewers’ comments” (E89).

Authors’ responses almost fully confirm publishers’ claims, with 166 of 170 authors (98%) confirming that they received reviews. The other four authors (2%) denied that they received reviews. Three publishers published monographs from these authors: two university publishers and one commercial publisher. Thus, it can be stated that authors’ responses confirm that analyzed publishers control the peer-review process by sending reviews to authors.

### S5: Publishers ask reviewers for permission to publish their names

We assumed that publishers that use open-identity labels ask reviewers for permission to publish their names. Such permission granted by reviewers can be interpreted as conveying manuscript approval.

Only four of 20 publishers said they asked for permission, but all analyzed publishers publish reviewers’ names on the editorial pages of monographs. Furthermore, according to these publishers, they actually did not ask for permission, but merely included information about publishing reviewers’ names in the contracts. This practice is illustrated by the following statement: “It is a part of the contract. We do not present reviewers’ names without a contract. We just do not use such reviewers” (E123). Another publisher stressed that asking for permission is important when using personal data: “In the cover letter sent to reviewers, we write–I think it is important because of personal data protection–that the name of (the) reviewer will be presented on the editorial page, so that the reviewer knows about it” (E113). Our analysis shows that publishing reviewers’ names on monographs is not connected with asking for permission to do it. Publishers that do not ask for permission refer to social practices in the academic community. They believe that it is obvious for reviewers that reviewing manuscripts is directly connected to publishing reviewers’ names on the editorial pages. For instance, one publisher said: “We do not ask because it is obvious for us that if a reviewer is an academic staff member and agrees to review, they know that this information about reviewing will be presented in the book” (E177). Another publisher stressed: “When a reviewer agrees to make a review, then he or she agrees to present their names as a mark of the publication quality on the editorial page” (E129).

Although most publishers do not ask reviewers for permission to publish their names, the publication of reviewers’ names can be identified as a mark of publication quality. All publishers said that they publish reviewers’ names only when reviewers approve manuscripts for publication. For instance, one interviewee mentioned that when “reviewers accept the manuscript, then we present their names” (E116). Another interviewee stressed that reviewers’ names on the editorial page provide evidence that “a monograph has been accepted for publication, so this monograph is reliable, can be cited, and used as a reference book” (E17).

We have verified whether reviewers’ responses confirm publishers’ claims. Table 2 presents reviewers’ responses to the question of whether publishers asked them for permission to publish their names. These responses are correlated with corresponding publishers’ claims.

**Table 2:** A comparison of reviewers’ responses (*n* = 212) when asked whether publishers seek their permission to publish their names, with corresponding publishers’ claims.

	Number of publishers	Publishers’ perspective	Reviewers’ perspective				Total number of responses	
			Asked		Not asked			
			n	%	n	%	n	%
	4	Ask	31	84	6	16	37	100
	16	Not ask	145	83	30	17	175	100
Total	20	–	176	83	36	17	212	100

Although most publishers said that they did not ask reviewers for permission to publish their names, reviewers' responses indicated otherwise. However, this discrepancy might be due to vagueness concerning how asking for permission is understood by publishers and reviewers. 176 (83%) reviewers said that publishers asked them before publishing their names on editorial pages, whereas only 36 (17%) reviewers said they did not. Thus, publishers’ claims do not correspond with reviewers’ responses. The permission that reviewers provide is not significantly related to publishers’ claims that they ask for permission (χ^2^ = .02, *p* > .05). The vast majority of reviewers said that they had given publishers permission both in the group of publishers that said they obtained permission and in the group of publishers that said they did not ask for permission.

Differences between reviewers and publishers’ responses can indicate a different understanding between these groups as to what it means to grant and obtain permission to publish reviewers’ names. It might result from different forms of giving and obtaining permission (written contracts, email, oral assent) and different timing when asking (before or after reviewing).

### S6: Publishers archive documents produced in a peer-review process

We assumed that publishers using open-identity labels archive documents produced during the peer-review process. All publishers said they archived reviews and other documents (e.g., contracts with authors and reviewers). All documents from particular peer-review processes are archived in a dedicated folder for that particular published item. The following statements illustrate this practice: “Each monograph has its own folder (…) In this folder, there are all documents connected with publishing this monograph: from the very beginning up to the publication itself” (E224); “all reviews (are) archived in the dedicated folder” (E237). All publishers claimed that all documents are accessible for at least three years after publishing the monographs, but most publishers stressed that documents are archived forever: “Those folders are not deleted after the publishing process, but they are archived, so it is easy to use them at any moment” (E223); “documents related to publications (are) assigned the A category at our publishing house, which means they are stored for an indefinite period of time” (E214).

## Discussion

In this paper, we examined whether *open-identity labels* used for published scholarly books can be understood to be a type of peer-review label. It should be noted that we analyzed only single-authored monographs, but open-identity labels are used for all types of published scholarly books. Furthermore, Poland’s labeling process does not differentiate by scholarly-book type, nor by number of authors.

We studied the editorial and reviewing practices of 20 of the 40 largest Polish publishers of scholarly books, examining publishers’ perspectives on how they work with authors and reviewers.

Our study reveals that the *open-identity label* in Poland is not formalized like in Flanders and Finland. Nevertheless, these practices are similar, and both types of labels confirm that a given scholarly book was peer-reviewed (Statement 1) and that Ph.D.-level reviewers or other experts from the pertinent field approved the work for publication (Statement 2). The reviewer-approval process before publication–corresponding with Finnish and Flemish standards–is common for all analyzed publishers despite there being no obligation to use it in Poland. Thus, an open-identity label used in Poland can be a delineation criterion for a research-evaluation system, and as such, is a type of peer-review label.

The lack of a formalized procedure at the national level, like in Finland and Flanders, results in various practices related to controlling the peer-review process. For instance, providing explicit criteria to reviewers (Statement 3) does not always take place. However, Finland’s label requirements state that the review process needs to focus on scientific quality, but it is not clear how publishers communicate this to referees. In Finland and Flanders, the peer-review label–like disclosing reviewers’ names in Poland–is not a formal requirement for performance-based funding distribution or evaluation, but it is closely aligned with it.

Generally, manuscripts were reviewed more frequently by one reviewer (54% of manuscripts) than two reviewers or more (46% of manuscripts). This distinguishes the Polish practice from the Finnish and Flemish requirements, in which two reviews are required. Polish university publishers said that when they seek a reviewer, it is always an external reviewer (not an academic staff member of a given university). Thus, this dimension of the Polish practice does not fully correspond with Finnish and Flemish regulations. Nonetheless, Polish publishers always send reviews to authors (Statement 4), as do Finnish and Flemish publishers that use peer-review labels.

Publishers in Finland and Flanders must meet various requirements to use peer-review labels and apply them to external organizations that control the use of labels. In Poland, using open-identity labels is a social practice that is treated as an internal part of the whole process of reviewing (Statement 5). Polish publishers assume that reviewers are aware that being a reviewer means also giving publishers permission to disclose the reviewer’s name. In addition, an unwritten rule indicates that only the names of reviewers who accept manuscripts for publications are disclosed. Moreover, publishers publish only manuscripts with positive reviews. Nonetheless, no data are available about rejections of poor manuscripts. All documents related to the review of a given manuscript are archived (Statement 6) and accessible even after a few years. Thus, these labelling elements correspond with Finnish and Flemish regulations.

Peer-review labels can elicit various side effects. For instance, Borghart [14] argues that in Flanders, publishers’ reputations were replaced by a standardized procedure that the academic community does not always welcome. Such a procedure is useful for assessing scholarly books within a national-level evaluation or funding system as an alternative to citation-based tools like Web of Science or Scopus. However, two issues of concern should be noted. First, this solution is very local and problematic because it does not include books published outside a given country. Second, Borghart [14] argues that using a peer-review label does not necessarily improve manuscript assessments that publishers conduct. Thus, a peer-review label might be perceived as not being the best indicator of the publishers’ prestige. However, one can claim that the label does not necessarily need to be perceived as a prestige indicator. For instance, in Finland, publishers, which are categorized on all levels of the authority list of book publishers (a higher level represents higher prestige), use the label. Moreover, Baldwin [23] argues that some standardization procedures, e.g., the U.S. grant system, have been implemented to ensure fairness to researchers and their careers/reputations. In light of such objectives, standardization of the book assessment also can be perceived as a process to ensure fairness.

Open identities are not perceived necessarily as a welcome direction in opening the peer-review process. Ross-Hellauer et al. [17] show that disclosing reviewers’ names can lead to reviewers being less critical. However, they also report that open identities can increase review quality. However, as Polka et al. [16] stress, open identities could lead to retaliation from authors. Thus, they argue that open identities should be optional, not mandatory. Baldwin [23] argues that a challenge to reviewers’ open identities was presented during a discussion on peer review in the 1970s at the National Science Foundation (NSF), a U.S. science agency. The NSF supported anonymous peer review to ensure scientific excellence; however, critics of the NSF policy argued that reviewers’ names should be publicly available. Critics felt that the peer-review process should be subject to public accountability. Furthermore, Biagioli [24] shows that hiding these referees’ identities in scientific publications creates the perception that they are “nameless voices of a geographically dispersed and multicentered scientific field” (p. 34). Thus, open identities can be viewed as a practice that focuses more on public accountability than on scientific quality.

The open-identity label is common across the Polish publishing landscape for scholarly books (e.g., universities, commercial publishers, learned societies et al.). However, on the basis of our study, it is not possible to say how well such practices among the largest, most prestigious publishing houses represent the other end of the spectrum, comprising very small, local publishers that only occasionally publish scholarly books.

While evaluation standards and practices usually seem to stem from journal-publishing cultures within the publishing industry, the open-identity label could be an interesting solution that might work in both cultures. However, it should be noted that the practice of open-identity labeling in Central and Eastern Europe’s book-publishing industry was not inspired by open peer review in journal publishing. It is an older practice whose roots have not been studied fully yet. Nonetheless, considering that this practice is visible in Central and Eastern Europe, rather than in Western European countries, one can conclude that it is part of the communist or socialist heritage. Despite the fact that almost three decades have passed from the breakdown of communist rule in Central and Eastern Europe, in academia, some practices remain rooted in past values. In Poland, each document needs to be signed and stamped by a few top-level management officers. For instance, in academia, a standard invoice for printer paper must be signed by six or seven different officers and researchers (including the principal investigator, the head of the institute, the dean, and the principal financial officer). Thus, the sender and the content of such a document very often are much less important than the person who countersigned the document. It is not an efficient way of completing tasks, but it allows institutions to blur the lines of responsibility. In light of this argument, disclosing reviewers’ names also can be perceived as part of a similar practice. Book authors can suggest and publishers can choose eminent scholars as reviewers to show that a given book has been approved by scholars who are well-known in the field. Thus, although publishers are the actual gatekeepers, they can shift responsibility for books’ scientific quality to reviewers.

The open-identity label also might be understood as an obstacle in selecting the best reviewers. During the Congress of the Polish Academic Publishers in 2018, this study’s primary results were presented by one of its authors. Discussing the findings, some heads and directors of publishing houses suggested that the open-identity label should be transformed into a peer-review label similar to that of Finland and Flanders. The key argument for this was not disclosing reviewers’ names in those countries. In Poland, when reviewers’ names and scientific degrees are disclosed, some publishers do not select the best experts in the field when an expert has “only a Ph.D.,” whereas a book author is a full professor.

## Conclusions

Most importantly, our analysis shows that an open-identity label can be used as a criterion for delineating peer-reviewed scholarly publications, and as such, it is a type of peer-review label similar to the Finnish and Flemish instruments. Moreover, our findings show that peer review of scholarly books before publication is standardized to some extent, despite the fact that no official regulations exist to administer it.

This paper shows that disclosing reviewers’ names is part of actual peer review of scholarly books and that both authors and reviewers have verified publishers’ stated practices. It is worth considering whether it would benefit the Polish open-identity label to formalize the practice, at least to some extent, as in Finland and Flanders. Formalization of the procedure by publishers themselves or learned societies could promote and help improve peer-review standards among publishers that have not yet adopted such sound practices. Nonetheless, peer review of scholarly books in Poland might be perceived as somehow formalized because publishers sign contracts with reviewers, the overwhelming majority of whom are paid for their reviews.

In line with our study, we suggest using the *open-identity label* as a delineation criterion in book-evaluation systems in countries where such labels and systems are operated. We believe that such labels can be used, but only as one of many indicators in evaluation and funding systems that produce scholarly books.

## Appendix 1

Topics and sample questions for interviews

### Category 1. Characteristics of Scholarly Publishers

**Topic 1.1.** Publisher profile

1. Please specify your publishing profile.
2. What types of books do you publish?
3. In which disciplines does your publishing house specialize?
4. Are you open to publishing scholarly books in all disciplines?
5. Who can publish a book with you?
6. *This question is only for university publishers*: Are you open to publishing scholarly books by authors who are not part of the academic staff at your university?

**Topic 1.2.** Publishing advisory boards

1. What competencies do editors have?
2. What duties do editors have?
3. Does your publishing house have a publishing advisory board?
4. What are the competencies for publishing advisory board members?
5. What work does the publishing advisory board do?

### Category II. Evaluation of submitted manuscripts

**Topic 2.1.** Manuscript-submission process

1. Should authors submit book proposals or complete manuscripts?
2. How does a manuscript get submitted and published in your publishing house?
3. *This question is only for university publishers*: Are manuscripts submitted by departments or by authors themselves?

**Topic 2.2.** Evaluation of manuscripts

1. What scholarly books do you accept for publication?
2. How do you evaluate scholarly books?
3. What criteria do you use to evaluate a scholarly book?
4. Are books assessed differently based on discipline? If so, what are these differences?
5. How do you evaluate whether a book is a scholarly book?
6. Do you provide book-evaluation rules on your website?
7. Do you have policies for dealing with unethical practices?
8. Do you make these policies available on a website?
9. Do you have explicit rules of conduct and consequences within the publishing house when unethical practices are discovered?
10. What percentage of scholarly books is rejected before being submitted for the review process? Can you describe typical rejected books?

**Topic 2.3.** Peer review

1. Who reviews book manuscripts for you?
2. How many referees review a manuscript? What criteria are used in such decisions?
3. How do you select reviewers?
4. *This question is only for university publishers*: Do you employ reviewers who are not academic staff members of your university?
5. What do you expect from reviewers?
6. What aspects of the manuscript do you think reviewers should evaluate?
7. What is the form of the review?
8. Do you use a questionnaire? What does it ask?
9. What features do you think a well-prepared review should contain?
10. What happens to each review’s results? Please describe how you utilize review results.
11. Do you send review reports to authors?
12. What happens if reviews are contradictory, e.g., one is positive and another is negative?
13. Do authors revise their manuscripts in response to reviews?
14. How do you deal with manuscripts that are the basis for academic promotions, i.e., habilitation books and professorial books?
15. What percentage of reviews was negative in the previous year?
16. What percentage of manuscripts was rejected due to negative reviews in the previous year?

**Topic 2.4**. **Archiving data on book reviews**

1. Does the publishing house archive reviews in such a way that they are available for viewing after three years?
2. What data about the peer-review process can be retrieved after three years?

**Topic 2.5**. **Reviewers’ names**

1. Do you include reviewers' names in all scholarly books?
2. Where do you place reviewers' names in books?
3. What is the purpose of this information?
4. How do you decide about the order of reviewers if two or more are listed?
5. Do you ask reviewers whether they agree to their names being disclosed in books that they review?

**Topic 2.6**. **Rejections**

1. What percentage of manuscripts was rejected without sending them to reviewers in the previous year? Can you characterize these books?
2. What percentage of reviews was negative in the previous year?
3. What percentage of manuscripts was rejected because of negative reviews in the previous year?

### Category III. Publishing market

**Topic 3.1**. **Market value of books**

1. What role in the decision to publish a scholarly book does its market value, i.e., potential profits from sales, play?
2. Do you offer royalties to authors?
3. Do you offer royalties to reviewers?
4. Do authors need to pay for book publication?
5. *If the answer to Question 4 is in the affirmative*: What are the sources of authors’ financial contributions?

**Topic 3.2**. **Changes in scholarly-book market**

I would like to ask three more questions about changes that have occurred in recent years.

1. Have you noticed some change in authors’ attitudes toward publishing scholarly books in your publishing house?
2. Have you noticed any changes in reviewers’ approach to evaluating scholarly books in your publishing house?
3. Have you noticed any changes in the scholarly-book market?

## Conclusion

1. Would you like to add something to our interview that I have not touched upon?

## Appendix 2

Questionnaire 1: Authors

All questions relate to a monograph you published in the [PUBLISHER NAME] during the years 2013–2016. If you published more than one monograph during these years, please provide answers on the latest one.

### Monograph

1. What area of knowledge does your monograph represent (in the case of many areas, please select the most suitable one)?

Please choose one of the following answers

❏ Natural sciences
❏ Engineering and technology
❏ Medical and health sciences
❏ Agricultural sciences
❏ Social sciences
❏ Humanities

2. Was your monograph related to academic promotion?

Please choose one of the following answers

❏ Yes
❏ No

[If the answer to Question 2 is “Yes,” another section of the questionnaire will be made available.]

3. How was your monograph related to academic promotion?

Please choose one of the following answers

❏ It was the basis for the habilitation procedure
❏ It was the basis for awarding the title of professor
❏ Other:

### Evaluation of Monograph

4. Did you send a book proposal to [PUBLISHER NAME]?

Please choose one of the following answers

❏ Yes
❏ No

5. Did the publishing house initially assess your monograph’s manuscript before the review process?

Please choose one of the following answers

❏ Yes
❏ No

6. Was your manuscript peer-reviewed?

Please choose one of the following answers

❏ Yes
❏ No
❏ I do not know

[If the answer to Question 6 is “Yes,” another section of the questionnaire will be made available.]

7. Did you provide the publisher with potential reviewers’ names?

Please choose one of the following answers

❏ Yes
❏ No

8. How many reviewers evaluated your manuscript?

Please choose one of the following answers

❏ 1
❏ 2
❏ 3
❏ 4
❏ 5 or more
❏ I do not know

9. Have you received any review(s)?

Please choose one of the following answers

❏ Yes
❏ No

[If the answers to Questions 6 and 9 are “Yes,” a further section of the questionnaire will be made available.]

10. What form did the review(s) take?

Please choose one of the following answers

❏ Descriptive form
❏ Questionnaire without descriptive part
❏ Questionnaire with descriptive part
❏ Other:

11. Did the review(s) contain an explicit conclusion?

Please choose one of the following answers

❏ Yes
❏ No

12. Did you need to revise the manuscript while considering all or some comments from reviewers?

Please choose one of the following answers

❏ Yes
❏ No

13. Did you need to prepare a written response to review(s)?

Please choose one of the following answers

❏ Yes
❏ No

14. Was the manuscript submitted to reviewers for re-evaluation?

Please choose one of the following answers

❏ Yes
❏ No
❏ I do not know

15. In your opinion, was/were the review(s) reliable?

Please choose one of the following answers

❏ Yes
❏ No
❏ Other:

### Author

16. Please indicate your gender:

Please choose one of the following answers

❏ Female
❏ Male

17. Please indicate the degree/title that you held at the time of publishing this monograph

Please choose one of the following answers

❏ Ph.D.
❏ Ph.D. habil.
❏ Professor
❏ Not applicable
❏ Other:

### Comments

18. Other comments:

Thank you for completing the survey.

## Appendix 3

Questionnaire 2: Reviewers

All questions relate to a monograph that you reviewed for the [PUBLISHER NAME] and was published during the years 2013–2016. If you reviewed more than one monograph from these years, please provide answers on the latest one.

### Monograph

1. What area of knowledge does a monograph reviewed by you represent (in case of many areas, please select one that is the most suitable)?

Please choose one of the following answers

❏ Natural sciences
❏ Engineering and technology
❏ Medical and health sciences
❏ Agricultural sciences
❏ Social sciences
❏ Humanities

2. Was this monograph related to academic promotion of the author?

Please choose one of the following answers

❏ Yes
❏ No
❏ I do not know

[If the answer to Question 2 is “Yes,” a further section of the questionnaire will be made available.]

3. How is this monograph related to academic promotion?

Please choose one of the following answers

❏ It was the basis for the habilitation procedure
❏ It was the basis for awarding the title of professor
❏ Other:

### Evaluation of Monograph

4. How do you assess your qualifications to review this monograph (matching the topic of this monograph)?

Please choose one of the following answers

❏ Very high
❏ High
❏ Medium
❏ Low
❏ Very low

5. How many reviewers evaluated the manuscript?

Please choose one of the following answers

❏ 1
❏ 2
❏ 3
❏ 4
❏ 5 or more
❏ I do not know

6. What form did the review(s) take?

Please choose one of the following answers

❏ Descriptive form
❏ Questionnaire without descriptive part
❏ Questionnaire with descriptive part
❏ Other:

7. Did the publisher provide evaluation criteria?

Please choose one of the following answers

❏ Yes
❏ No

8. Did the publisher expect an explicit conclusion?

Please choose one of the following answers

❏ Yes
❏ No

9. How did you rate the manuscript?

Please choose one of the following answers

❏ Recommendation for publication
❏ Recommendation for publication after revisions
❏ Recommendation for rejection
❏ Other:

10. Did you receive a response from the author concerning the review?

Please choose one of the following answers

❏ Yes
❏ No

11. Did you receive a revised version of the monograph for re-evaluation?

Please choose one of the following answers

❏ Yes
❏ No

12. Did you give the publisher your permission to place your name on the monograph’s editorial page?

Please choose one of the following answers

❏ Yes
❏ No

[If the answer to Question 12 is “Yes,” a further section of the questionnaire will be made available.]

13. In what form did you agree to include your name on the monograph’s editorial page?

Please choose one of the following answers

❏ Orally
❏ E-mail communication
❏ Written contract
❏ Other:

### Reviewer

14. Please indicate your gender:

Please choose one of the following answers

❏ Female
❏ Male

15. Please indicate the degree/title that you had at the time of reviewing this monograph:

Please choose one of the following answers

❏ Ph.D.
❏ Ph.D. habil.
❏ Professor
❏ Not applicable
❏ Other:

### Comments

16. Other comments:

Thank you for completing the survey.

## Supporting information

S1 File Dataset B1 (Authors) from the integrated quantitative and qualitative data. (XLSX)

S2 File Dataset B2 (Reviewers) from the integrated quantitative and qualitative data. (XLSX)

## References

[1] Björk B-C, Solomon D. The publishing delay in scholarly peer-reviewed journals. J Informetr. 2013;7(4): 914–923. 10.1016/j.joi.2013.09.001

[2] Marsh HW, Jayasinghe UW, Bond NW. Improving the peer-review process for grant applications: Reliability, validity, bias, and generalizability. Am Psychol. 2008;63(3): 160–168. 10.1037/0003-066X.63.3.160 18377106

[3] Giménez-Toledo E, Sivertsen G, Mañana-Rodríguez J. Peer review as a delineation criterion in data sources for the assessment and measurement of scholarly book publishing in social sciences and humanities. 16th International Conference on Scientometrics and Informetrics, ISSI 2017. 2017. pp. 118–124.

[4] Giménez-Toledo E, Mañana-Rodríguez J, Sivertsen G. Scholarly book publishing: Its information sources for evaluation in the social sciences and humanities. Res Eval. 2017;26(2): 91–101. 10.1093/reseval/rvx007

[5] Langfeldt L. The policy challenges of peer review: Managing bias, conflict of interests and interdisciplinary assessments. Res Eval. 2006;15(1): 31–41. 10.3152/147154406781776039

[6] Daniel H-D. Guardians of science: Fairness and reliability of peer review Weinheim, Germany: VCH Verlagsgesellschaft; 1993.

[7] König T. Peer Review in the Social Sciences and Humanities at the European Level: The Experiences of the European Research Council In: Ochsner M, Hug SE, Hans-Dieter D, editors. Research Assessment in the Humanities: Towards Criteria and Procedures. Cham, Switzerland: Springer International Publishing; 2016 pp. 151–163. 10.1007/978-3-319-29016-4_12

[8] Spezi V, Wakeling S, Pinfield S, Fry J, Creaser C, Willett P. “Let the community decide”? The vision and reality of soundness-only peer review in open-access mega-journals. J Doc. 2018;74(1): 137–61. 10.1108/JD-06-2017-0092

[9] Pölönen J. Applications of, and Experiences with, the Norwegian Model in Finland. Journal of Data and Information Science. 2018;3(4): 30–43. 10.2478/jdis-2018-0019

[10] Giménez-Toledo E, Mañana-Rodríguez J, Engels TCE, Guns R, Kulczycki E, Ochsner M et al Taking scholarly books into account, Part II: A comparison of 19 European countries in evaluation and funding. Scientometrics. 2018 10.1007/s11192-018-2956-7

[11] Kulczycki E. The diversity of monographs: Changing landscape of book evaluation in Poland. Aslib J Inf Manag. 2018;70(6): 608–622. 10.1108/AJIM-03-2018-0062

[12] Pölönen J, Engels TCE, Guns R, Verleysen FT. Is my publication peer reviewed? A comparison of top-down and bottom-up identification of peer review in the framework of the Finnish and Flemish performance-based research funding systems. Science, Technology and Innovation indicators STI 2017. Paris, France; 2017.

[13] Verleysen FT, Engels TCE. A label for peer-reviewed books. J Am Soc Inf Sci Technol. 2013;64(2): 428–430. 10.1002/asi.22836

[14] Borghart P. A label for peer-reviewed books? Some critical reflections. Learn Publ. 2013;26(3): 167–171. 10.1087/20130303

[15] Engels TCE, Guns R. The Flemish performance-based research funding system: A unique variant of the Norwegian model. Journal of Data and Information Science. 2018;3(4): 44–59. 10.2478/jdis-2018-0020

[16] Polka JK, Kiley R, Konforti B, Stern B, Vale RD. Publish peer reviews. Nat. 2018;560: 545–547. 10.1038/d41586-018-06032-w 30158621

[17] Ross-Hellauer T, Deppe A, Schmidt B. Survey on open peer review: Attitudes and experience amongst editors, authors and reviewers. PLoS One. 2017;12(12): e0189311 10.1371/journal.pone.0189311 29236721 PMC5728564

[18] Creswell J. Research Design: Qualitative, Quantitative, and Mixed-Methods Approaches Thousand Oaks, CA: Sage; 2014.

[19] Bernard HR. Research methods in anthropology: Qualitative and quantitative approaches 4th ed Lanham, MD: AltaMira; 2006.

[20] Saldaña J. The Coding Manual for Qualitative Researchers Los Angeles, CA: SAGE; 2009 10.1017/CBO9781107415324.004

[21] Fetters MD, Curry LA, Creswell JW. Achieving integration in mixed-methods designs: Principles and practices. Health Serv Res. 2013;48(6pt2): 2134–2156. 10.1111/1475-6773.12117 24279835 PMC4097839

[22] Fetters MD, Freshwater D. Publishing a Methodological Mixed Methods Research Article. J Mix Methods Res. 2015;9(3): 203–213. 10.1177/1558689815594687

[23] Baldwin M. Scientific Autonomy, Public Accountability, and the Rise of “Peer Review” in the Cold War United States. Isis. 2018;109(3): 538–558.

[24] Biagioli M. From Book Censorship to Academic Peer Review. Emergences: Journal for the Study of Media & Composite Cultures. 2002;12(1): 11–45. 10.1080/1045722022000003435

